# Physical assessment, spectroscopic and chemometric analysis of starch-based foils with selected functional additives

**DOI:** 10.1371/journal.pone.0212070

**Published:** 2019-02-13

**Authors:** Tomasz Oniszczuk, Maciej Combrzyński, Arkadiusz Matwijczuk, Anna Oniszczuk, Bożena Gładyszewska, Janusz Podleśny, Grzegorz Czernel, Dariusz Karcz, Agnieszka Niemczynowicz, Agnieszka Wójtowicz

**Affiliations:** 1 Department of Thermal Technology and Food Process Engineering, University of Life Sciences in Lublin, Lublin, Poland; 2 Department of Physics, University of Life Sciences in Lublin, Lublin, Poland; 3 Department of Inorganic Chemistry, Medical University in Lublin, Lublin, Poland; 4 Institute of Soil Science and Plant Cultivation—State Research Institute, Puławy, Poland; 5 Department of Analytical Chemistry, Faculty of Chemical Engineering and Technology, Cracow University of Technology, Krakow, Poland; 6 Department of Analysis and Differential Equations, Faculty of Mathematics and Computer Science, University of Warmia and Mazury, Olsztyn, Poland; Cranfield University, UNITED KINGDOM

## Abstract

The paper presents the results of studies related to the impact of functional additives in the form of polylactide (PLA), polyvinyl alcohol (PVA), and keratin hydrolysate (K) on the physical characteristics of biopolymer foils. TPS granulate was obtained using a TS-45 single-screw extruder with L/D = 16. Foil was produced with the use of an L/D = 36 extruder with film-blowing section. The impact of the quantity and type of the functional additives on the processing efficiency and energy consumption of granulate extrusion, as well as the physical characteristics of the foil produced: thickness, basis weight, and colour were determined. By measuring the FTIR spectra it was determined the type and origin of the respective functional groups. It was observed that foils produced from granulates with the addition of 3% PVA were characterised by the lowest thickness and basis weight. Addition of 2 and 3% of PLA increased thickness and basis weight of starch-based foils significantly. Increasing the content of keratin in SG/K samples resulted in a decrease of brightness and intensify the yellow tint of foils, especially when 2 and 3% of keratin was used. In terms of the other samples, it was observed that the colour remained almost unchanged irrespective of the percentage content of the additive used. Infrared analyses conducted on foil containing PVA, PLA, and K revealed a change in spectra intensity in the frequency range associated with–OH groups originating from the forming free, intra- and intermolecular hydrogen bonds. Based on an analysis of the respective bands within the IR range it was also concluded that considerable structural changes took place with respect to the glycosidic bonds of starch itself. The application of the mentioned additives had a significant structural impact on the produced starch-based foils. Furthermore, the conducted UV-Vis analyses revealed a substantial increase in absorbance and a related reduction of the permeability (colour change) of the obtained materials in the range of ultraviolet and visible light.

## Introduction

In recent years, due to the fast progressing depletion of global oil reserves as well as the fact that petroleum-based materials are non-biodegradable, efforts are increasingly being made to obtain more environmentally friendly (biodegradable) packaging materials. The development of mass production procedures for biodegradable materials remains the subject of extensive research conducted both by academics and at various industrial laboratories [1–3]. For several decades, plastic materials have been employed in nearly every area of our everyday lives, including packaging (e.g. foils, bags, bottles, etc.), construction (various elements of interior design, windows, etc.), medicine, transport, electronics, or agriculture [4]. Indeed, it would now be hard to imagine a life entirely devoid of the plastic found in our packaging, toys, cars, medical devices, etc. [5–7]. However, apart from the undeniable benefits of its use, plastic is also a source of many problems. It is becoming increasingly difficult to manage the bulk of used plastic packaging produced in the conventional manner and the situation weighs heavily on the condition of our natural environment and the health of humans and animals alike.

Numerous studied are now conducted in this context with the view of obtaining biodegradable foils and other similar products from renewable materials. This research is aimed at developing the optimum methods for extruding foil from natural materials so as to obtain high-quality products that would be in no way mechanically inferior to those obtained from plastic. Such materials can hope to find broad use in, for instance, agriculture, e.g. for mulching, composting or protection of soil and plants [8]. Particular attention has recently been paid to a variety of naturally obtained, starch-based materials [9–11]. However, for unprocessed starch to be usable as a biodegradable material, it must first be transformed into thermoplastic starch (TPS). The biopolymer is obtained from starch mixed with a plasticizer–usually glycerine or water, to allow liquification of the material in the process of extrusion at a temperature lower than the temperature of starch decomposition [12–15]. Unfortunately, the thus obtained pure thermoplastic starch comes with a number of significant drawbacks, including: low mechanical strength (brittleness) and high susceptibility to environmental factors such as humidity [16–18]. At the same time, however, starch provides a number of significant benefits, including: high biodegradability, availability, relatively low production cost, and exceptional chemical modifiability [19]. It is required that biodegradable polymer foils provide functional characteristics comparable to those offered by traditional packing foils. This can be accomplished by developing better production methods and selecting adequate functional additives [20, 21]. It should be emphasised that the replacement of conventional plastics (e.g. in agriculture) with biodegradable materials will not reduce the amount of waste material but will instead provide mankind with alternative means of waste processing by way of organic recycling. Another innovative approach may also involve the use of protein or lipids obtained from agricultural sources in the production of biodegradable materials (aside from the far more commonly used carbohydrate-based raw materials). In order to eliminate the weaknesses of TPS-based foils, their production could be supplemented with various polymers compatible with starch, for instance polyvinyl alcohol (PVA), PLA or keratin, which can alter the mechanical characteristics of the obtained biopolymers [22–24].

Starch is a natural carbohydrate consisting mainly of glucose mers, with a 20 to 25% content of amylase and 75 to 80% content of amylopectin joined by (1→4)-α-D-glycosidic and (1→6)-α-D-glycosidic bonds. PLA (polylactic acid, polylactide) is a polymer from the group of aphylactic polyesters. It is fully biodegradable. It can be obtained from renewable natural raw materials such as cornmeal or other cereals [25]. Keratin–is a group of fibrillar proteins insoluble in water and produced by keratinocytes. PVA—poly(vinyl alcohol) is a vinyl polymer. Formally, it is a polymer of vinyl alcohol, but it is in fact obtained from poly(vinyl acetate). It is used e.g. as a component in glues, varnishes, textile apprets, in the production of chemical apparatus, as an emulsion paint stabiliser, pharmaceutic thickener, in the production of protective gloves, surgical sutures, foils, petrol-resistant plates and pipes, oils, and various fibres [24].

The aim of the study was to evaluate selected physical properties and to perform spectroscopic analyses of potato starch-based TPS foils with various functional additives.

The study determined the influence of various amounts and types of functional additives on the efficiency and energy consumption of the process of extruding thermoplastic starch granulate, as well as selected physical characteristics of the obtained starch foils, i.e.: thickness, basis weight, and colour. In order to determine the impact of the additives on the possibility of affecting the material’s molecular structure, we employed the method of UV-Vis spectroscopy (mainly with the view of determining changes on permeability by radiation within the UV and Vis ranges) and especially the method of FTIR spectroscopy [26, 27]. On the basis of FTIR spectra measurement, we identified the respective bands and associated them with vibrations of the particular functional groups [6]. The research discussed in this paper was also aimed at correlating the respective changes in the physical qualities of the analysed materials (induced by the use of the aforementioned additives) with changes in their FTIR spectra, which could potentially also allow the identification of their respective spectroscopic markers. Moreover, it was very important to determine the type of molecular level interactions between starch and the applied additives, as continued use of such materials would entail exposure to e.g. UV radiation, which could affect the chemical structure of the macromolecules, altering foil characteristics and potentially reducing its usable lifetime. It is noteworthy that the optical methods employed in this study, specifically UV-Vis spectroscopy and FTIR spectroscopy, are fast, cost-effective, and most importantly non-destructive.

## Materials and methods

### Materials

The primary raw materials used in the production of thermoplastic starch granulates (TPS), from which biodegradable foils were subsequently extruded, were: “Superior” potato starch (ZPZ, Łomża) with the moisture content of 14.9%, crude glycerol (ZPCH Odczynniki Chemiczne, Lublin, Poland), poly(vinyl alcohol) (PVA) with the molecular mass of 72000 and hydrolysis rate of 82–89% (POCH S.A, Poland), polylactide (PLA) Ingeo Biopolymer 2003D (NatureWorks LLC, Minnetonka, USA), and keratin hydrolysate powder (Proteina Wytwórnia Naturalnych Białek S.C., Łódź, Poland). The exact composition of mixtures used in the study is presented in Table 1.

**Table 1:** Composition of TPS granulates used for blowing extrusion of biodegradable foil, % by mass.

Sample	Potato starch	Glycerol	PVA	PLA	Keratin
SG/PVA	79.0	20.0	1.0	0	0
	78.0	20.0	2.0	0	0
	77.0	20.0	3.0	0	0
SG/PLA	79.0	20.0	0	1.0	0
	77.0	20.0	0	2.0	0
	75.0	20.0	0	3.0	0
SG/K	79.0	20.0	0	0	1.0
	78.0	20.0	0	0	2.0
	77.0	20.0	0	0	3.0

The proper amount of dry components was mixed for 5 min, than glycerol was added and blends were mixed using a laboratory-grade ribbon mixer for 15 min, and left in tightly sealed plastic bags for 24 h to homogenise the blends. Then the prepared material was again mixed for 10 minutes immediately prior to extrusion, which guaranteed the looseness and uniformity of the mixture.

## Methods

The process of extrusion of the TPS granulate was conducted using a modified TS-45 single-screw extruder (Z.M.Ch. Metalchem, Gliwice, Poland) with L/D = 16. An extrusion die with a single, φ = 3 mm hole was used. The extrusion process was performed at the temperature of 70–90°C in the respective sections of the extruder, and the extruder screw speed was set to 80 rpm. The thermoplastic starch granulate obtained in the process of extrusion was dried using a laboratory-grade air dryer at 50°C until the material moisture content was reduced to 4–6%. After drying, the granulate was stored in sealed containers.

### Processing efficiency and energy consumption of TPS granulate production

Efficiency of the extrusion process was measured by determining the mass of the thermoplastic starch granulate that was produced in a given period of time, and comparing the same between the respective analysed mixtures. The test was performed in 3 replicates for each series, and the mean from three measurements was considered in calculating the final result. The efficiency was calculated as proposed by Matysiak et al. [28]:

$$Q=\frac{m}{t}\left(\frac{kg}{h}\right)$$ (1)

where: Q–processing efficiency (kg/h),

m–mass of the extrudate (kg),

t–time (h).

The energy consumption of the process of extruding thermoplastic starch granulate was determined with the use of a wattmeter connected to the extruder’s motor. The parameters of the motor’s operation were considered, including its load and efficiency measured for the respective replicates. The energy consumption was measured three times and calculated as the specific mechanical energy (SME) consumption according to Ryu and Ng [2001]:

$$SME=\frac{n}{n_{m}}\times \frac{L}{100}\times \frac{P}{Q}\left(\frac{RWL}{kg}\right)$$ (2)

where: SME–specific mechanical energy (kWh/kg), n–screw speed (rpm), n_m_−maximum screw speed (rpm), L–engine load (%), P–power (W), Q–processing efficiency (kg/h).

### Processing of TPS foil

The obtained starch granulates containing PVA, PLA and keratin at various percentages were used to produce foil using a film-blowing extruder machine with L/D = 36 adequately modified for this purpose at the Department of Thermal Technology and Food Process Engineering University of Life Sciences in Lublin (by SAVO, Wiązowna, Poland). The modification entailed a specific configuration of the screw and the plasticization unit of the extruder as well as including an additional compressed air-cooling unit on the head [29]. In the course of film-blowing extrusion, a foil sleeve was obtained with the use of the extrusion forming die head with a 60 mm orifice and 0.8 mm working aperture. The extrusion temperature, depending on the section of the extruder, was within the range from 72 to 125°C. The temperature of the die was maintained at 110–115°C. The speed of the extruder screw was approx. 80 rpm.

### TPS foil thickness and basis weight

Foil thickness was determined with the use of a micrometer. 10 samples of 10x10 cm were cut out from the foil produced from the TPS granulates containing the respective additives [29]. Measurements were taken in 3 places for each of the samples.

Basis weight corresponds to the weight of one square meter of the given product. For the purposes of this measurement, 10 samples of 10x10 cm were cut out from the produced foil sleeves. The prepared samples were weighed using an analytical balance (precision up to 0.001 g). The basis weight was calculated from the following formula:

$$B_{w}=\frac{M}{S}\left(\frac{g}{m^{2}}\right)$$ (3)

where: B_w_−basis weight (g/m^2^), M–mass of the sample (g), S–sample size (m^2^).

### Colour profile of starch-based foils

Colour characteristics of foil based on TPS starch with various additives was evaluated for surface colour profile measurements (sample 10x10 cm) using Lovibond CAM-System 500 Colour and Appearance Measurements System (The Tintometer UK). CIE-Lab scale was applied for the evaluation of L* for brightness (0–100), a* (+) for redness and (-) for greenness, and b* (+) for yellowness and (-) for blueness, accordingly. Colour checking was performed in 10 replications for each sample. ΔE*_ab_ was calculated as a colour change index [30]. Background was taken as reference value with L* = 92.0. a* = -0.4. b* = -2.0, when calculating the ΔE*_ab_ values.

### Electronic absorption spectroscopy (UV-Vis)

Electronic absorption spectra were recorded at 23°C on a double-beam UV-Vis spectrophotometer Cary 300 Bio (Varian, USA) equipped with a thermostatted cuvette holder with a 6x6 multicell Peltier block. The temperature was controlled with a thermocouple probe (Cary Series II, Varian, USA) placed directly in the quartz cuvette. The spectra were recorded from 180 to 800 nm. Absorbancy was measured on film samples with dimensions of 10x50 mm.

### FTIR spectroscopy

The measurements of ATR-FTIR background-corrected spectra were carried out in solvents using a ZnSe trough (45° cut, yielding 10 internal reflections) crystal plate for liquids and were recorded with a Vector 3300 spectrometer (Bruker Optik GmbH, Germany). Typically, 25 scans were collected, Fourier-transformed, and averaged for each measurement. The IR absorption spectra at a resolution of one data point per 1 cm^-1^ were obtained in the region between 4000 and 400 cm^-1^. The instrument was purged with argon for 40 min before and then during the measurements. The ZnSe crystal was cleaned with ultra-pure organic solvents (*Sigma-Aldrich* Co.). All experiments were carried out at 23°C.

### Principal Component Analysis

One of the most famous mathematical tools for reducing of the dataset is Principal Component Analysis (PCA), which is probably used in all scientific areas. Its goal is to extract the important information from the dataset and to express this information in the terms of new orthogonal variables called principal components (PCs). This variables are the linear combinations of the original variables. The largest possible variance is captured by the first principal component. In order to obtain the PCs we decompose of the dataset matrix X according the following rule:

$$X=FQ^{T}$$ (4)

where the matrix *F* denotes the matrix of factor scores and the matrix *Q* gives the coefficients of the linear combinations used to compute the factors scores. Principal Component Analysis of fluorescence spectra were performed using OriginPro 2018 Programme using the Principal Component Analysis for Spectroscopy App.

## Results and discussion

### Results of processing efficiency and energy consumption during processing of TPS granulates

Table 2 presents the results of efficiency and energy consumption measurements for the extrusion processing of potato-starch-based TPS granulates with various functional additives. It was observed that the efficiency and energy consumption results were significantly dependent on the uniformity of raw material fed into the devices cylinder and the fluidity of material flow through the extrusion die.

**Table 2:** Results of processing efficiency and energy consumption during extrusion of TPS granulates.

Sample	Additive amount (%)	Efficiency (kg/h)		SME (kWh/kg)	
		mean	SD	mean	SD
	1	18.2	0.5292	0.042	0.0012
**SG/PVA**	2	18.6	0.3464	0.040	0.0007
	3	19.2	0.4000	0.037	0.0008
	1	17.2	0.3464	0.048	0.0010
**SG/PLA**	2	16.2	0.6928	0.049	0.0022
	3	14.6	0.8718	0.055	0.0032
**SG/K**	1	21.2	0.9165	0.035	0.0015
	2	22.2	0.6110	0.033	0.0009
	3	22.4	0.7211	0.030	0.0010

In the case of PVA used as the functional additive, it was observed that varying amount of the additive used had a significant effect on the efficiency of the extrusion process. In the case of this additive, the highest efficiency was recorded for the highest PVA content in the material mixture. Polyvinyl alcohol caused structural loosening of the mixture, which facilitated easier feeding and flow of the material through the extruder. The lowest efficiency was observed for mixtures containing PLA, respectively from 14.6 to 17.2 kg/h, where the highest efficiency of the extrusion process correlated with the lowest content of the additive. The overall best processing efficiency was observed for mixtures containing keratin, with the extrusion efficiency improving with the increasing keratin content. The highest efficiency was observed for the mixture containing 3% of keratin. At the same time, however, the overall differences observed between the respective measurements were not significant, as evidenced by the low values of standard deviation obtained from the calculations of processing efficiency for the respective mixtures, irrespective of the type or amount of additive used (Table 2).

It is noteworthy at this point that the energy consumption of the extrusion process is the primary factor determining the economic viability of the production process. It was observed in the case of PVA, similarly to the parallel processing efficiency measurements, that changing additive content correlated to changes of the power consumption ratio. For this particular additive, the lowest energy consumption was observed for the highest PVA content in the mixture. The power consumption recorded for the extrusion of mixtures containing PLA was the highest of all values obtained during the tests and was, respectively, from 0.048 to 0.055 kWh/kg, with the lowest value corresponding to the lowest content of the additive. Conversely, the lowest energy consumption values were observed for mixtures containing keratin, of which the overall lowest value was recorded while processing the SG/K3 mixture containing 3% keratin.

### Results of TPS foil thickness and basis weight measurements

Foil thickness is one of the primary indicators of its quality, as well as the quality and uniformity of the processing machine’s operation. In industrial conditions, thickness measurements of foil obtained through film-blowing extrusion are registered continuously as a part of the production process, so any deviations with regard of the material thickness are carefully analysed in terms of film-blowing quality and uniformity. Table 3 presents the results of foils thickness measurements after processing of starch-based TPS granulates containing the respective functional additives. Thickness of foils obtained with the use of the laboratory-grade extruder were largely dependent on the uniformity of the granulate feed, fluidity of the material flow through the extrusion die and intensity of film-blowing relative to the amount and pressure of the compressed air fed to the inner ring of the extruding head during formation the foil sleeve. No explicit influence of varying additives concentration on the measured basis weight of starch-based foils was observed. In the case of SG/PVA samples, the lowest thickness was noted for the highest PVA concentration in the mixture. Polyvinyl alcohol loosened the material structure and increased its elasticity, which allowed for easier film-blowing with the use of compressed air and therefore produced thinner foil. The thickness of starch foil produced from SG/PLA granulates was overall the highest, reaching between 0.136 and 0.199 mm with the lowest thickness observed for foils with the lowest additive content. Similarly, in the case of keratin, the lowest film thickness was observed in foil produced from SG/K granulate with the lowest content of the additive, while increasing the content of keratin in the mixture produced thicker foil which, however, tended to decrease slightly if the 3% was used in blend, both in the case of PLA and keratin. Generally, the results of foil thickness measurements were similar, and no significant discrepancies were observed in material cut out from the foil sleeves in various location, as evidenced by the low values of standard deviation, irrespective of the type and amount of the additive used.

**Table 3:** Results of TPS foil thickness and basis weight.

Sample	Additive amount (%)	Thickness (mm)		Basis weight (g/m^2^)	
		mean	SD	mean	SD
**SG/PVA**	1	0.126	0.010	148.08	11.76
	2	0.168	0.016	196.62	15.23
	3	0.119	0.011	133.21	13.58
**SG/PLA**	1	0.136	0.013	150.84	20.16
	2	0.199	0.039	242.14	48.97
	3	0.189	0.038	216.99	31.26
**SG/K**	1	0.122	0.010	138.34	15.18
	2	0.152	0.014	181.71	16.30
	3	0.145	0.013	170.66	20.18

Basis weight is a property inherently related to foil thickness and it is, among other applications, used as the basis for packing material classification. When determining the basis weight of starch-based foils containing the respective functional additives, similar correlations to those noted in material thickness measurements were observed. The lowest basis weight was obtained for foil produced from the SG/PVA granulate with the 3% content of polyvinyl alcohol in the mixture. Conversely, the highest basis weight was registered for foil obtained from SG/PLA granulate with 2 and 3% content of PLA in the mixture. Foils with higher basis weight are typically used for industrial and agricultural purposes where small differences in terms of grammage within a given type of foil are less important qualitatively than the mechanical properties and biodegradability of the material.

Needless to say that the relationship between the amount of added additives (resulting in a change in basis weight or film thickness) is reflected in the FTIR spectra (see below in the text). It can be observed measurable shift in the position of the peaks of FTIR spectras, as have been explained in details in 3.4 section.

### Results of TPS foil colour profile

Measurements of the respective colour factors for starch-based foil correlated with the use of the respective functional additives. The mean values of the respective colour factors are presented in Table 4. The greatest diversification of colour, both in terms of brightness L* and chromaticity coordinates a* and b*, was observed when analysing starch-based foil produced from SG/K granulates containing between 1 and 3% of keratin in the material mixture. We also observed a reduction of the greening hue of expressed in the negative values of colour a* which was shifted from negative values noted for 1% and 2% content of the additive to positive values resulting in the emergence of a reddish hue (positive values of the chromaticity coordinate a*) for 3% content of keratin in the material mixture. The foils produced from SG/PVA and SG/PLA granulates showed consistently similar values of the a* coordinate. In terms of the measurement of the colour chromaticity coordinate b*, negative values were observed, indicating the presence of a blueish hue, in foils produced with the addition of polyvinyl alcohol, although increasing the additive content did not produce an apparent colour factor for the colour b*. While measuring colour parameters for foil containing PLA, we observed a slight increase of brightness L* and reduction of the chromaticity of colour b* signalling stronger yellow hue of the foil with the increasing content of the PLA additive. In the case of foil produced from the SG/PLA granulate, the differences in terms of colour between the respective PLA additive concentrations were slight but all the foils returned the value of ΔE*_ab_ above 2.3, which indicates the possibility of visually distinguishing the foil colour from a background with defined colour factors. ΔE*_ab_ is a single number that represents the 'distance' between two colours in CIE-Lab colour scale. For values of ΔE*_ab_ below 2.3, the colour difference is imperceptible for the human eye, hence the value is often referred to as the JND threshold JND (just noticeable difference), whereas values over 2.3 are discernible, but for the range 3.5 < ΔE < 5 the clear difference in color is noticed [31]. The addition of keratin also yielded significant correlations between the chromaticity coordinate b* and the content of the additive; the higher the amount of keratin in the SG/K granulate, the more intensive the foil’s yellow hue would become. The differences could even be perceived visually, which is related to the high values of the colour change index (distance metric) ΔE*_ab_ whose values were from 3.19 to 9.61, whereby the change was visibly discernible and related to the characteristics of the keratin additive used.

**Table 4:** Results of TPS film colour profile.

Sample	Additive amount (%)	L*		a*		b*		ΔE*_ab_
		mean	SD	mean	SD	mean	SD	
**SG/PVA**	1	90.20	0.44	-1.04	0.32	-1.16	0.08	1.33
	2	89.80	0.51	-1.12	0.16	-0.56	0.32	2.01
	3	89.60	0.40	-1.36	0.32	-1.04	0.32	1.95
**SG/PLA**	1	88.28	0.30	-1.20	0.08	0.32	0.16	3.66
	2	89.13	0.68	-1.33	0.30	0.28	0.15	3.09
	3	89.87	0.14	-1.33	0.29	0.13	0.37	2.59
**SG/K**	1	89.16	0.32	-1.20	0.16	0.48	0.16	3.19
	2	87.23	0.19	-1.07	0.30	2.35	0.35	5.79
	3	83.57	0.79	0.17	0.36	4.07	0.36	9.61

L*—brightness; a*—redness-greenness balance; b*—yellowness-blueness balance; ΔE*_ab_*—*colour change index

### Results of spectroscopic analysis of TPS foil with various additives

Altering the composition of thermoplastic foil with the addition of PVA, PLA or keratin affects the thermomechanical properties of the material and therefore has a bearing on both the molecular structure and the morphology of the polymer, increasing its elasticity and improving the functional viability of materials obtained from such mixtures [32, 33].

#### Results of TPS film UV-Vis spectroscopy

**Fig 1:**
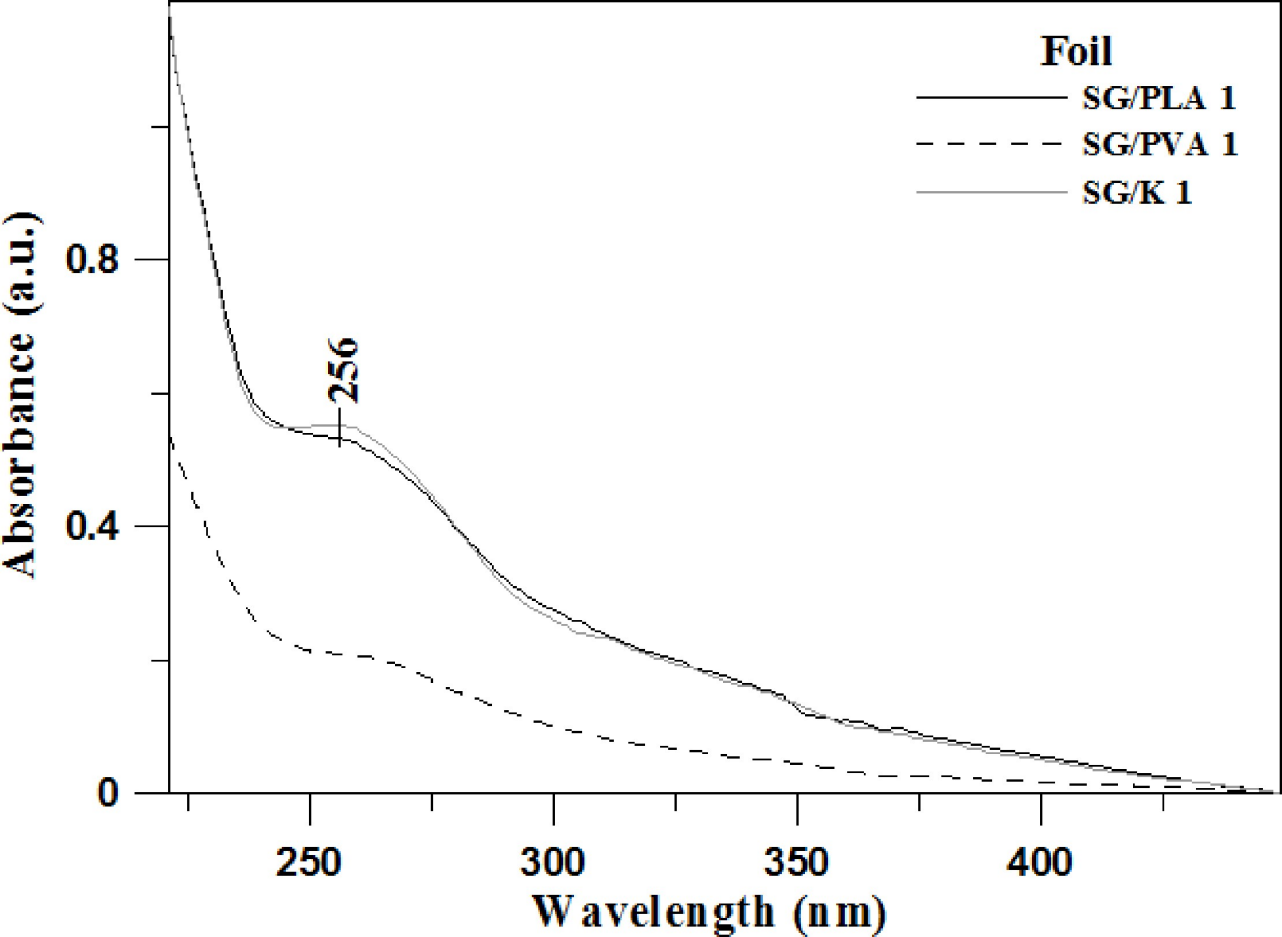
Electronic absorption spectra of the foils selected for the study: SG/PLA (solid black line), SG/PVA (dashed black line), SG/K (solid grey line).

Fig 1 presents the electronic absorption spectra for the respective analysed foils: SG/PLA, SG/PVA and SG/K. For ease of interpretation, in each case the presented spectra correspond only to a 1% content of the respective additive. Where larger quantities of additives were used (2, 3%), the electronic absorption spectra did not differ significantly in shape, only in intensity, when compared to the presented bands (S1 Fig). The locations of the observed absorption maxima, cantered at approx. 260 nm in all studied materials (256 nm) clearly indicate a n→π* electronic transition in the carbonyl group (C = O), which may be taking place in the applied additives (under the influence of degradation processes or as a direct result of applying the additive).

What is particularly noteworthy, however, is the presence of the aforementioned broad absorption region located between ~ 230 nm and 350 nm (and significantly weaker but still visible between 350 and 400 nm) in the electronic absorption spectra for all the studied materials. The presence of this region clearly evidences the high quality of the obtained materials with regard to the transmission of the entire visible spectrum and absorption of only UV radiation. The rate of absorption is naturally increased with the growing content of the additives used. Studies conducted for different percentage amounts of the functional additives (S1 Fig) returned results similar to the presented spectra of mixtures containing 1% of the respective additive.

#### Results of TPS film FTIR spectroscopy

The method of Fourier-transform infrared spectroscopy (FTIR) is currently increasingly used in compound analyses, particularly in materials containing biodegradable additives (thermoplastic starch, potato starch, crude glycerol, polyvinyl alcohol (PVA), polylactide (PLA), keratin hydrolysate, and many others), as well as for the purposes of measuring their content in various product whose functionality they may affect. The possibility of using FTIR for determining the content of the aforementioned compounds in biodegradable materials has been confirmed by a growing number of researchers and is, among other factors, due to the fact that the absorption of certain functional groups, e.g. the band of the carbonyl group C = O (1700–1800 cm^-1^, formed e.g. as a result of degradation processes taking place in this type of materials or due to the content of various additives) in the starch fraction of the analysed product is relative weak or non-existent, while addition of various additives tends to quickly and significantly increase the intensity of absorption in this region. Moreover, starch itself gives off very intensive and interesting infrared bands as, since it is a carbohydrate (plant polysaccharide), it is composed exclusively of glucose mers linked by α-glycosidic bonds. In fact, starch contains two fractions: unbranched amylose formed from glycose residues linked by oxygen atoms by way of α-1,4-glycosidic bonds, and branched amylopectin which contains additional α-1,6-glycosidic bonds. The use of the aforementioned additives creates intensive bands in the infrared range originating from the respective functional groups, which also suggests a significant modification of molecular interactions in the resulting foil group, as evidenced by the change in FTIR spectra of the materials selected for the tests [23].

**Fig 2:**
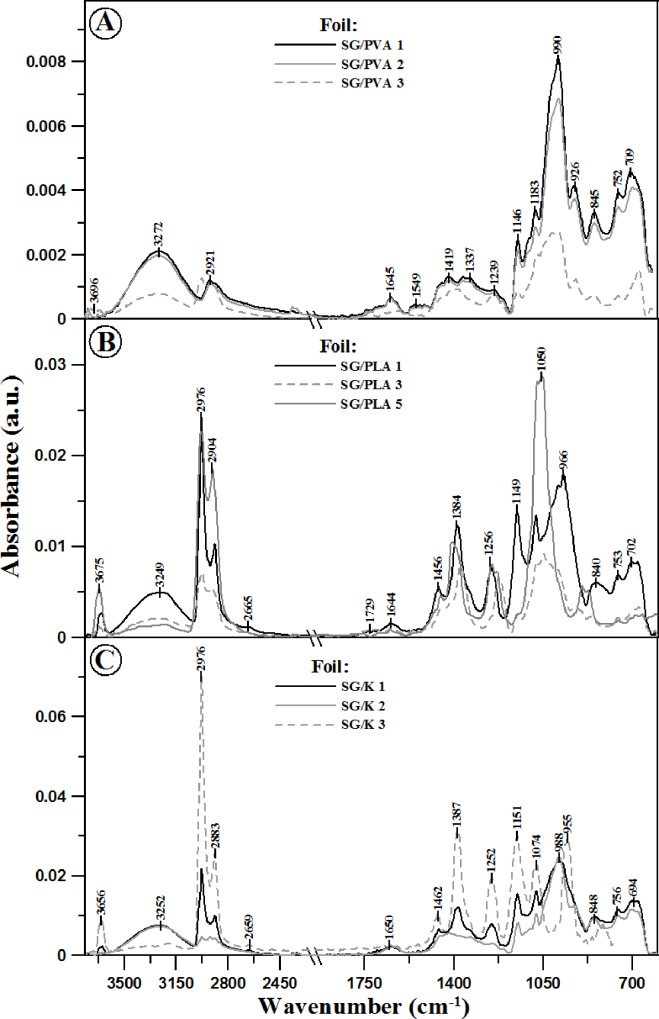
**ATR-FTIR absorption spectra for the analysed foils: SG/PVA (Panel A), SG/PLA (Panel B), SG/K (Panel C)**.

For easier analysis of the data, FTIR spectra in the respective panels were presented in Fig 2 whereas Table 5 presents all the vibrations observed in the studied samples together with the corresponding vibration bands of the particular functional groups [34–37]. It is immediately apparent that the use of the additives caused significant structural changes in the analysed foils. The changes are clearly evident also in the corresponding FTIR spectra reflecting strong intermolecular interactions taking place in the studied starch-based foils. Evidently, all the analysed samples contained a certain small amount of water, as reflected by the presence of the relatively intensive bands in the region of approx. 1640 cm^-1^ related to deformation vibrations of OH—δ_m_(O-H) (absorber water) and vibrations in the region 3600–3000 cm^-1^ with the maximum at ~ 3200 cm^-1^ belonging to OH stretching vibrations, υ(-OH) with absorber water. Vibrations with the maximum at approx. 1640 cm^-1^ may partially overshadow vibrations originating from the material from which the analysed foils were made. On the other hand, vibrations from the region of 3600–3000 cm^-1^, i.e. the stretching vibrations of OH groups, may originate from free, both intra- and intermolecular hydrogen bonds present in the starch structure. With the increasing percentage content of the additive, the formation of hydrogen bonds can modify the foil’s internal structure and therefore significantly modify the character of the observed vibrations. Another very important are of vibrations is found at 3000–2800, i.e. the region corresponding to C-H stretching vibrations originating from CH_2_ groups. The aforementioned vibrations with the maximum at approx. 1640 cm^-1^, i.e. -OH deformation vibrations, can also originate from the oscillations of water molecules bonded with starch which characterize 14.9% of moisture content before processing. In turn, the vibration region of 1300–1100 cm^-1^ belongs to the asymmetric stretching vibrations of C-O and can also originate from the vibrations of the C-O-C group; the vibrations are observed in natural polysaccharides. The region of 930–710 cm^-1^ can be correlated with the vibrations characteristic of the polysaccharide ring, i.e. vibrations belonging to the pyranose ring in particular units belonging to glucose itself.

**Table 5:** Positioning of the maxima of FTIR absorption spectra and assignment of the respective vibrations for selected test materials (biodegradable foils) within the spectral range of 3800–550 cm^-1^ [38–40].

Positioning of the maximum (cm^-1^)									Type and origin of vibrations
SG/PVA			SG/PLA			SG/K			
1	2	3	1	2	3	1	2	3	
3741	3742	3695	3658	3673	3666	3654	3650	3654	O-H^…^O-H
3265	3276	3274	3257	3451/3265	3474/3232	3271	3251	3199	υ(-OH) with absorber water
2920	2917	2980	2974	2980	2979	2991	2980	2979	
–	–	2903	–	–	–	2979/2972	2973	2971	υ(C-H)
–	–	–	–	2911	2903	2930	2919	2931	
2882	2891/2851	–	2888	2890	–	2887	2889/2851	2887	υ_st_(C-H) or υ(C-H) in CH_2_ / CH_3_ group
–	–	–	2666	–	2656	2668	2654	2660	overtone
–	–	1729	1725	1740	1755	1701	1699	–	υ(C = O)
1649	1645	–	1646	1648	1684/1648	1648	1645	1646	δ_m_(O-H) (absorber water)
1537	1539	1596	–	1541	–	–	–	–	δ(C-H) or δ(CH_2_) in plane
–	–	–	1460	1447	1450	1458	1454	1472	
1418	1416	1408	–	1410	1402	–	1419		C-H bending and wagging or δ(COH)
1362	1336	–	1383	1376	–	1383	–	1381	
–	–	–	1335	–	–	1338	1336	–	
1237	1240	1249	1252	1224	1253/1235	1253	1245	1251	δ(O-H) or C-O
1150	1149	1147	1152	1146	–	1148	1148	1150	anhydroglucose ring C–O stretch of C–O–H in starch and C-O-C antisymmetric bridge
1104/1078	1103/1077	1101/1074	1077	1075	1059	1077	1076	–	
1016	1009	1010	1014	1047	–	1115	–	1082/1072	υ (C-O) and υ(C-O-C or C-O-H)
–	–	–	–	1020	–	–	–	–	
997/986	990	986	985	984	–	987/973	987	964	
923	967/928	924	966	929	–	–	926	953	υ (C-C) and υ (C-O) or C-O-C bend or O-H deformation (broadened by water)
849	846	849	–	–	897	–	–	–	
–	–	762	842	861	870	848	852	834	
754	754	–	756	753	756	757	758	–	
705	701	–	701	–	–	700	706	–	
–	676	681	674	672	688	–	677	–	

υ–stretching, δ–deformation, s–symmetrical, as–asymmetrical, st–strong, m–medium

Within the range of wave numbers from 3600 to 3000 cm^-1^ (Fig 2) the characteristic broad band corresponding to the stretching vibrations of the -OH group showed the highest intensity in SG/K foil containing keratin, as compared to foils with other additives. Moreover, in this case the band maximum was significantly shifted towards higher wave numbers, which could suggest the formation of intermolecular hydrogen bonds as well as a higher content of water in the samples. This is corroborated by the increased intensity of the band related to the–OH deformation vibrations with the maximum at ~ 1640 cm^-1^ (as described above), related to the vibrations of starch itself. In the case of vibrations in the range of 3000–2800 cm^-1^ (characteristic of CH_2_ stretching vibrations), the most intensive bands were observed in the case of SG/PLA foil containing polylactide and SG/K foil containing keratin. This may be related, on the one hand, to the higher content of amylose and amylopectin in the studied material, and on the other, with stronger intermolecular interaction in the case of additives other than PVA. The FTIR spectra recorded for foils modified with all the considered additives revealed very significant changes in the spectra with the maximum at approx. 1250–70 and 950–1190 cm^-1^, i.e. in terms of vibrations related to skeletal vibrations of the C-O and C-C groups which are characteristic of starch itself. This could evidence, firstly, very good blending of the ingredients used in the preparation of the respective mixtures, and secondly, the existence of strong interactions between the same, manifested in the formation of strong hydrogen bonds between the respective groups. Below, at approx. 850 cm^-1^, we observed bands characteristic of α- glycosidic bonds typical of starch. In more detail, the spectral features observed in this region are related to conformational changes, which typically occur in starch at its α-glicoside bond.

In summary, it is also noteworthy that the applied additives very strongly modified the FTIR spectrum of the foil [41–43]. In the case of PVA, evident changes are observed for the content of 3%, whereas for PLA and K, the addition of any amount higher than 1% significantly modified the infrared spectrum of the analysed material. This can signify a strong impact of the additive on intermolecular interactions, both between starch molecules and between the molecules of starch and the applied additive [36].

### Results of Principal Component Analysis

**Fig 3:**
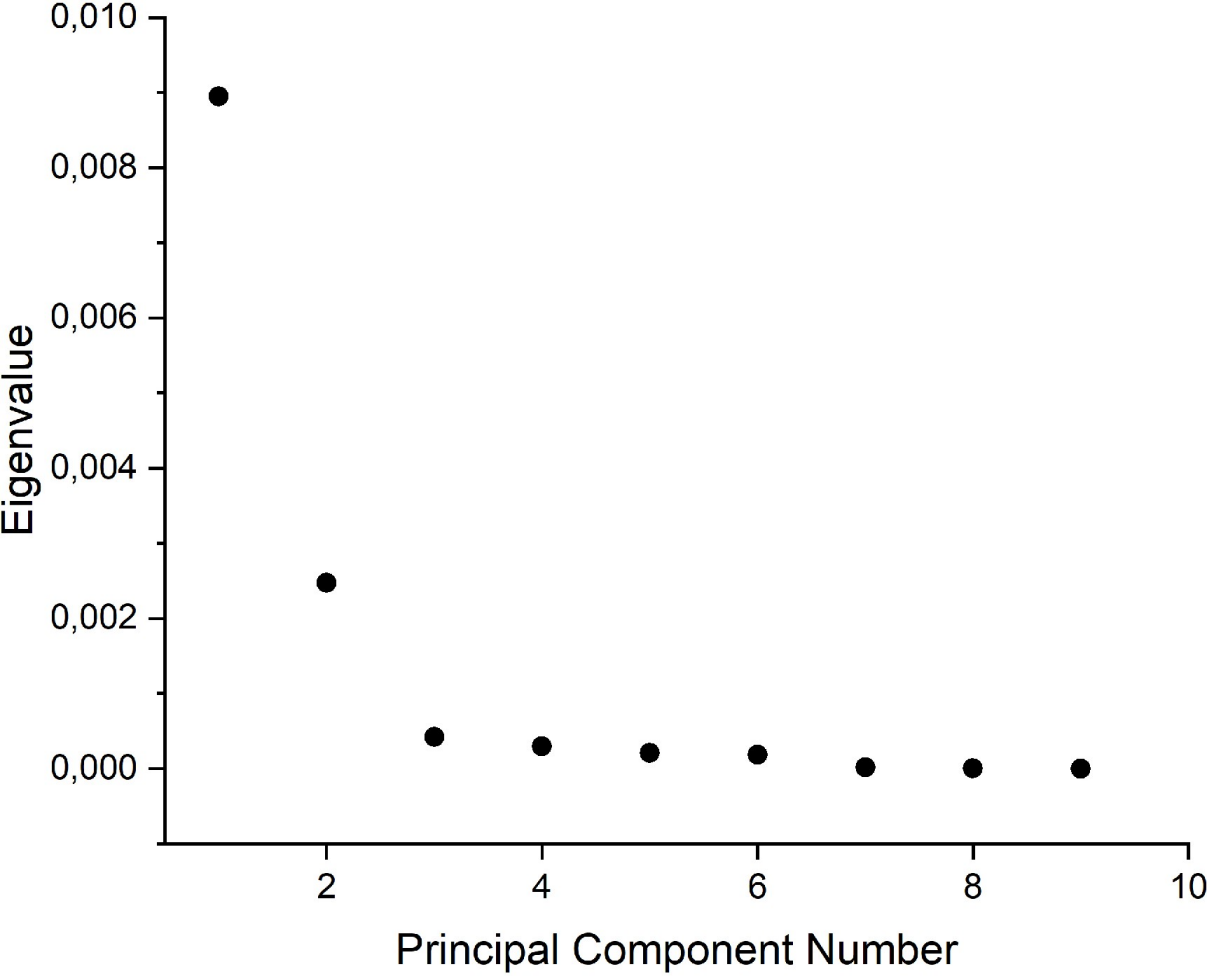
Contribution of eigenvalues with PCs.

In order to better understand the differences and similarities between the considered FTIR spectra and the influence of different compositions of foils on the same, we took advantage of the PCA multivariate technique. The aim of this analysis was to factorize the spectral data into several principal components embedding the spectral variations of each collected spectral data set. The most common features among the samples and their grouping are generally expressed by the first few PCs, with we identify based on scree test criterion (Fig 3). Samples with similar spectra tend to be grouped together in the score plot of the first two or three components.

**Fig 4:**
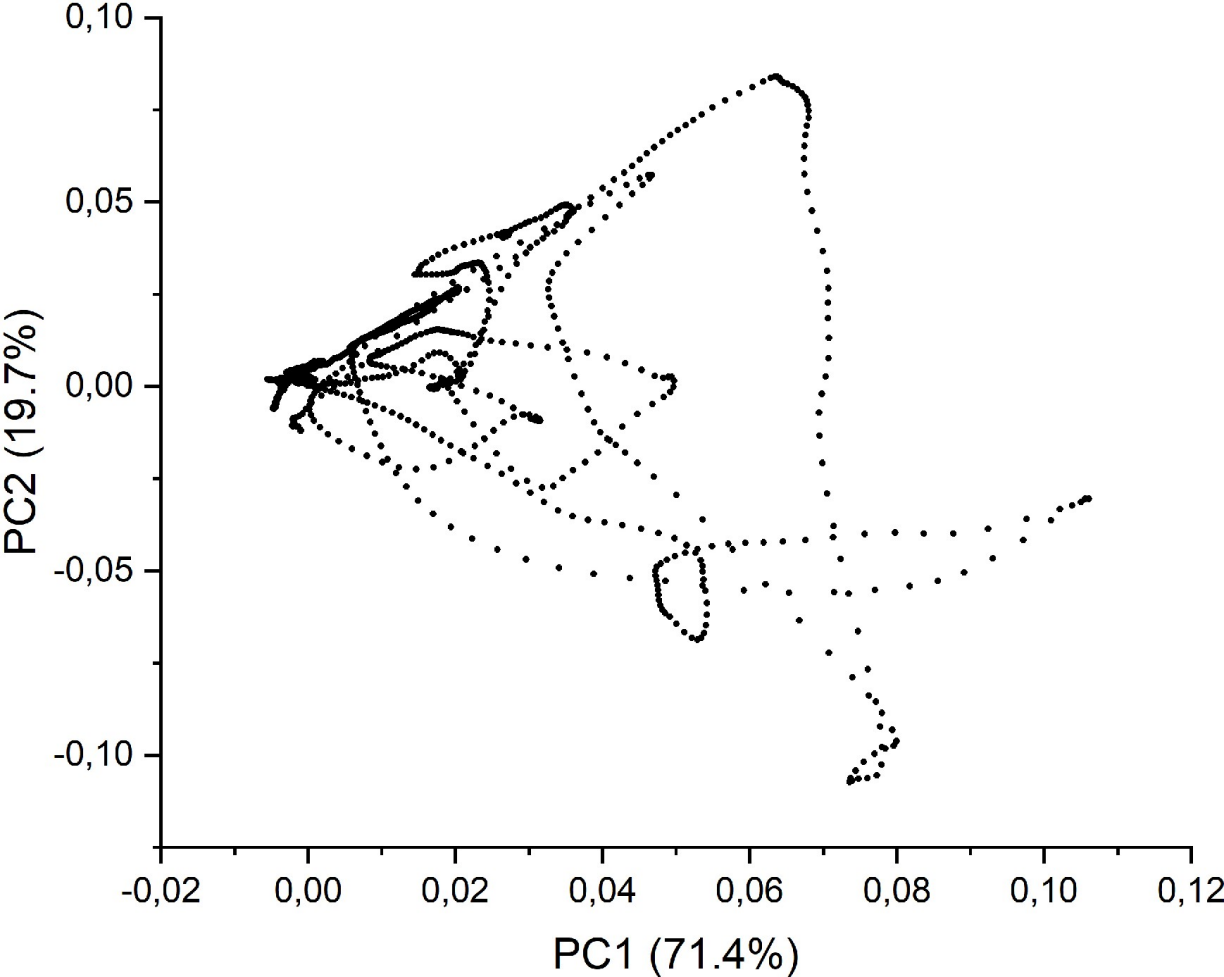
The loading plot from PCA.

**Fig 5:**
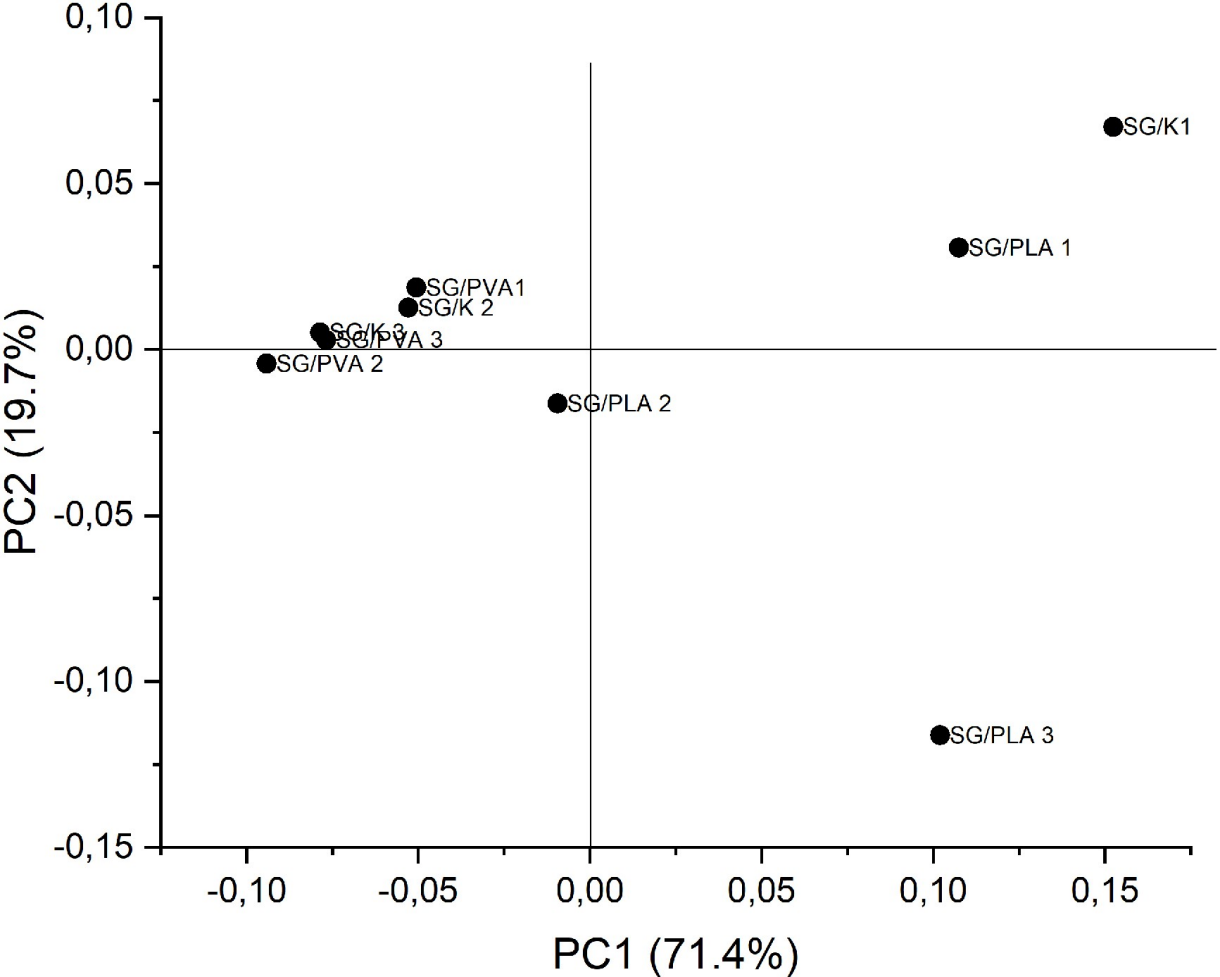
PCA score plot as 2D (PC1xPC2) derived from the FTIR spectra.

PC1 explained 71.4% of the total variance in the dataset while PC2 explained 19.7%. The contribution of the other PCs is shown in the Table 6. As it can be seen, a nearly complete description of the whole variance requires up to six PCs. In the first step, it was considered the contribution of the first two principal components as the ones most strongly influencing the total variance. The score and loading plot for PC1 vs. PC2 are shown in Fig 4 and Fig 5, respectively. Firstly, SG/PVA (1–3) and SG/K (1–2) are located exclusively in the left half of the plot (see Fig 5), while SG/K1 seems to be an outlier. On the other hand, for the positive values of PC1, SG/K1, SG/PLA1 and SG/PLA3 are located in the right-hand side half of the plot.

**Table 6:** Eigenvalues and variance percentage corresponding to the PCs.

Principal Component Number	Eigenvalue	Percentage of variance (%)	Cumulative (%)
**1**	0.00895	71.35713	71.35713
**2**	0.00247	19.71432	91.07145
**3**	4.17E-04	3.32573	94.39718
**4**	2.93E-04	2.33577	96.73295
**5**	2.10E-04	1.67166	98.40461
**6**	1.83E-04	1.46274	99.86734
**7**	1.32E-05	0.10527	99.97261
**8**	3.44E-06	0.02739	100
**9**	1.20E-33	9.60E-30	100

**Fig 6:**
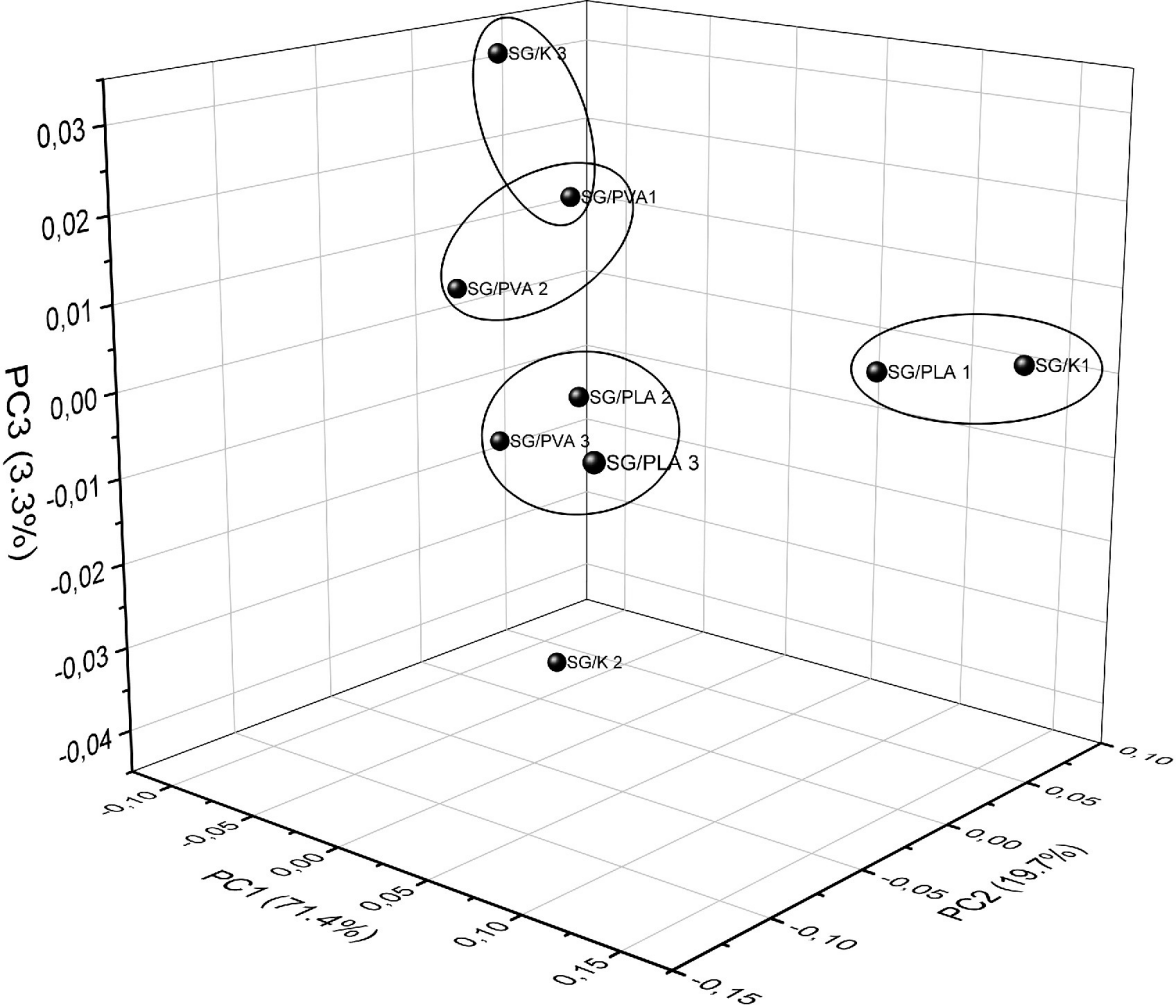
PCA score plot as 3D (PC1xPC2xPC3) derived from the FTIR spectra.

Using the plot in Fig 5, it is possible to partially suggest reasons for this location of the foils on the basis of their thickness, basis weight or another features. For example, the location of SG/PLA1 and SG/K1 in the upper right-hand quadrant of the score plot may be explained by the intensity of their FTIR spectra in the range 3000–2800 cm^-1^ and the location of the absorbance maxima in the UV-Vis spectra. Additionally, in the case of those foils the minimum mean values of basis weight were observed. Given influence of the third principal component (Fig 6) our research seems to confirm our assumption. In contrast, the SG/K2, SG/PVA2, and SG/PLA3 foils show the highest values of basis weight, so they must be located opposite SG/PLA 1 and SG/K1. An interesting factor influencing the location of foils in the score plot is the variety of colour factor. For example, it is easy to notice that SG/K1 foil, which returned the highest value of L* is situated in a quadrat of the plot opposite to that occupied by SG/K3, with the lowest value of L*. A similar situation is observed for the values b* in the group of SG/PLA foils. SG/PLA1 foil is situated in the upper right-hand quadrat of the plot, for PC1>0 and PC2>0, and has the highest value of factor b*. Conversely, SG/PLA3 foil is located in bottom right-hand quadrat of the plot, for PC1>0 and PC2<0, and has the lowest value of factor b*.

## Conclusions

The conducted study utilising UV-Vis electronic spectroscopy in the analysis of selected foils provided quick information on the transparency of the tested materials. It was demonstrated that the use of the respective materials significantly lowers foil permeability by UV and Vis radiation. The use of additives in SG/PVA, SG/PLA and SG/K foils significantly modified their functional characteristic and improved their ability to protect products that may be packed in the same.

On the other hand, the conducted FTIR spectroscopy analyses allowed us to qualitatively assess the impact of aforementioned additives on intercellular interactions, verify the potential changes in the amylose to amylopectin ratio in the starch itself, and indirectly provide a description of the extent to which the respective ingredients of the mixture had been blended during the preparatory stage.

## Supporting information

S1 Fig Representative electronic absorption spectra of SG/PLA foils with various percentages of additives: 1% (solid black line), 2% (dashed black line), 3% (solid grey line). (DOCX)
