# Supplementary material for: Physical assessment, spectroscopic and chemometric analysis of starch-based foils with selected functional additives
Source: PLoS One. 2019 Feb 13;14(2):e0212070. doi: 10.1371/journal.pone.0212070 (PMC6373948; doi:10.1371/journal.pone.0212070)
Supplement: S1 Fig — (DOCX) [file pone.0212070.s001.docx]

**Physical Assessment, Spectroscopic and Chemometric Analysis of Starch-Based Foils with Selected Functional Additives**

Tomasz Oniszczuk ^1^, Maciej Combrzyński ^1*^, Matwijczuk Arkadiusz^2*^, Anna Oniszczuk ^3^, Bożena Gładyszewska ^2^, Janusz Podleśny ^7^, Grzegorz Czernel ^2^, Dariusz Karcz.^5^, Agnieszka Niemczynowicz ^6^, Agnieszka Wójtowicz ^1^

^1^ Department of Thermal Technology and Food Process Engineering, University of Life Sciences in Lublin, Lublin, Poland

^2^ Department of Physics, University of Life Sciences in Lublin, Lublin, Poland

^3^ Department of Department of Inorganic Chemistry, Medical University in Lublin, Lublin, Poland

^4^ Department of Cell Biology, Institute of Biology, Maria Curie-Skłodowska University, Lublin, Poland
^5^ Department of Analytical Chemistry, Faculty of Chemical Engineering and Technology, Cracow University of Technology, Krakow, Poland

^6^ Department of Analysis and Differential Equations, Faculty of Mathematics and Computer Science, University of Warmia and Mazury, Olsztyn, Poland

^7^ Institute of Soil Science and Plant Cultivation - State Research Institute, Puławy, Poland

* Corresponding authors

E-mail: arkadiusz.matwijczuk@up.lublin.pl, maciej.combrzynski@up.lublin.pl





**S1 Fig**. Representative electronic absorption spectra of SG/PLA foils with various percentages of additives: 1% (solid black line), 2% (dashed black line), 3% (solid grey line).
